# An Itemized Bill

**Published:** 1871-01

**Authors:** 


An Itemized Bill.—A good anecdote is told of the famous French surgeon Nelaton. Going through one of the streets of Paris one day, he came upon a crowd standing in front of a drug store. There a man lay stretched out who had been terribly wounded by a sharp buggy shaft. His belly had been ripped and a large part of his intestines protruded. His life could be saved only by a very difficult and dangerous operation, but Nelaton was equal to the occasion, and soon his patient, quite a wealthy man, was sent home out of danger. For three weeks Nelaton heard nothing more of him, but then he made his appearance and asked his preserver how much he owed him. "Hundred and fifty francs," replied the surgeon. "That is much," said the man, "but give me a specified bill; here is your money." Nelaton sat down and wrote as follows: "For readjusting a yard and a half of the intestinal canal, at a hundred francs per yard, one hundred and fifty francs."

